# Clinical value of CT-guided percutaneous fine-needle aspiration biopsy for peritoneal lesions

**DOI:** 10.1186/s12880-020-00512-0

**Published:** 2020-11-02

**Authors:** Hualong Yu, Chuanyu Zhang, Shihe Liu, Gang Jiang, Shaoke Li, Liang Zhang, Yongjie Wang, Wenjian Xu

**Affiliations:** 1 Department of Radiology, The Affiliated Hospital of Qingdao University, Qingdao University, Jiangsu Road No. 16, Qingdao, 266101 Shandong People’s Republic of China; 2 Department of thoracic surgery,The Affiliated Hospital of Qingdao University, Qingdao University, Qingdao, 266101 Shandong People’s Republic of China

**Keywords:** Peritoneal, Computed tomography

## Abstract

**Background:**

To investigate the clinical value of CT-guided percutaneous fine-needle aspiration biopsy for peritoneal lesions of unknown nature.

**Methods:**

A retrospective analysis was conducted of 84 patients with peritoneal thickening for unknown reasons. There were 26 males and 58 females who underwent CT-guided percutaneous fine-needle aspiration biopsy for peritoneal lesions.

**Result:**

Among these 84 patients, no definite pathologic diagnosis was made in 3 patients, who were lost to the follow-up. The accuracy rate of CT-guided percutaneous fine-needle aspiration biopsy was 95.1% (77/81). Sixty lesions were pathologically-diagnosed with malignancies (74.1%), including 55 with peritoneal metastases, 4 with malignant mesotheliomas, and 1 with a lymphoma. Twenty-four patients (33.8%) were diagnosed as benign lesions, including 11 with tuberculosis and 13 with inflammatory lesions. The complications of CT-guided percutaneous fine-needle aspiration biopsy included bleeding in 1 patient and ascites leakage in 2 patients.

**Conclusion:**

CT-guided percutaneous fine-needle aspiration biopsy is a safe and effective method for diagnosing peritoneal lesions.

## Background

Peritoneal lesions have a variety of causes and the radiologic manifestations are complex and diverse. Routine clinical examination and radiologic examinations are of limited value for identifying the etiology and for differential diagnosis of malignant and benign lesions. Ultrasound-guided percutaneous fine-needle aspiration biopsy and laparoscopy is the conventional method to acquire peritoneal specimens for histopathologic evaluation; however, this procedure is more suitable for sampling of superficial peritoneal lesions and laparoscopy entails a high risk and cost. CT-guided percutaneous fine-needle aspiration biopsy has proven to be a mature and widely used technology [1], especially for lesions in the liver and pancreas. To date, few reports address CT-guided percutaneous fine-needle aspiration biopsy of the peritoneum. We conducted a retrospective analysis of 84 patients who underwent this procedure at our hospital in the past 4 years. The purpose of the study was to discuss the diagnostic value of this method for peritoneal lesions.

## Method

### Subjects

From January 2012 to December 2015, 84 patients undergoing CT guided percutaneous peritoneal puncture in our hospital were included in this study, including 26 males and 58 females (age range, 20–84 years).

### Instruments and equipment

A Philips Brilliance 16-slice CT scanner was used, with the following scan parameters: tube voltage, 120 kV; tube current, 150 mA; slice thickness, 3 mm; and pitch, 1.375. Two percent lidocaine was used as a local anesthetic. A BARD biopsy gun was used.

### Preparation before biopsy

The patients received a routine pre-operative ECG, and routine blood and coagulation function testing [2]. Informed consent for the biopsy was signed.

### Biopsy method

**Fig. 1:**
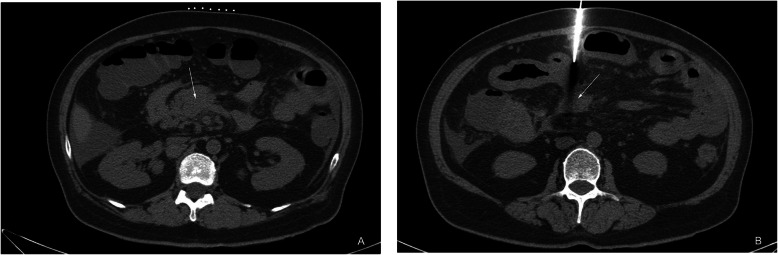
**a**, **b** One patient with gastric cancer. **a** Enlargement of mesenteric lymph nodes; **b** Adjacent intestinal canal was pushed using the sectioning method

The patient assumed a supine position. Based on historical radiologic data, a guide wire grid was placed on the body surface for localization, and the scan range was determined. Before surgery, the patients were trained to breathe at the correct amplitude. The puncture site was selected (typically the route shortest to the lesion of the omentum majus and away from the blood vessels and intestinal canal), and the distance from the lesion margin to the skin was measured. After local anesthesia was administered, the scan was performed for a second time to determine the puncture site and needle insertion scheme. Then, the biopsy was rapidly inserted to the lesion for sampling. If the lesion was closely related to the intestinal canal, a coaxial needle was used (Fig. 1). For each patient, 3–6 specimens were collected, fixed in formalin, and submitted to pathology. The diameter of the biopsy needle used in this study is 18G After the biopsy was performed, the incision was compressed for 10 mi n[3]. The patients were re-examined by local CT to observe whether or not there were complications, such as bleeding. Those patients without apparent abnormalities were instructed to lie in bed for 1–2 days. All cases were followed up 3 months, 6 months and 12 months after the completion of the biopsy to determine whether the results obtained by percutaneous puncture are consistent with the final clinical diagnosis, and to calculate the accuracy of the results of percutaneous biopsy.

## Results

### Histopathologic results of biopsy specimens

A total of 84 peritoneal lesions were obtained by fine-needle aspiration biopsy, all of which were successfully collected at the first attempt. Three patients were later lost to follow-up, and no final pathologic results were obtained. The specific pathologic types of the other 81 peritoneal lesions are shown in Table 1. The biopsy result was inconsistent with the final clinical diagnosis in 4 patients. Of 13 patients diagnosed with inflammatory lesions by biopsy, 1 was confirmed by surgery to have metastases from ovarian cancer. Three patients were clinically diagnosed with tuberculous peritonitis by combining exfoliative cytologic examination of peritoneal effusion, clinical manifestations, and experimental anti-tuberculosis therapy. The false negative rate was 4.9%(4/81),the false positive rate was 0. and the accuracy rate was 95.1%(77/81).

**Table 1:** Distribution of pathologic types according to CT-guided percutaneous fine-needle aspiration biopsy (case) (Three cases lost to follow-up were excluded)

Final diagnosis by biopsy	Positive for biopsy	Negative for biopsy
Metastases		
Metastases from ovarian cancer	43	1
Metastases from gastrointestinal cancer	5	0
Metastases from pancreatic cancer	1	0
Unidentified primary lesions	5	0
Malignant mesothelioma	4	0
Lymphoma	1	0
Pseudomyxoma peritonei	1	0
Tuberculous peritonitis	11	0
Inflammatory lesions	6	0
Total	80	4

### Postoperative complications

**Fig. 2:**
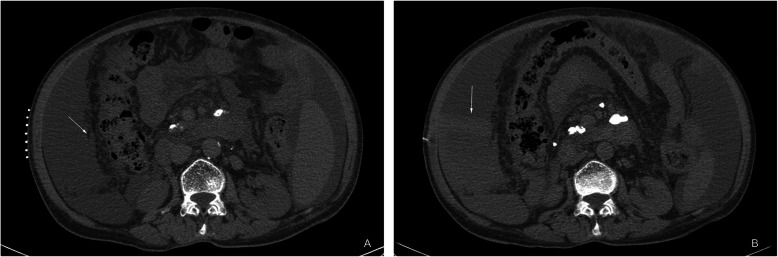
**a**, **b** One patient with ovarian cancer. **a** Thickening of the right omentum majus; **b** Active bleeding in the peritoneal cavity after biopsy

**Fig. 3:**
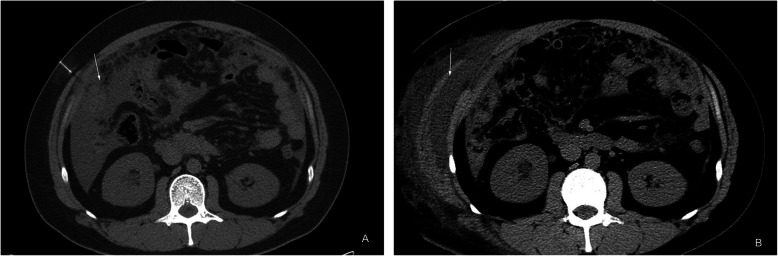
**a**, **b** One patient with tuberculosis. **a** Nodular peritoneal thickening; **b** Edema of the abdominal wall soft tissues by CT scan upon re-examination

In this study, 11 of 84 patients had complications, including hemorrhagic shock, pain, abdominal wall edema, swelling, etc. The incidence was 13.1%(11/84). The serious complication in this study was bleeding. One patient (1.2%) (Fig. 2):had shock-related symptoms due to bleeding, such as fatigue, cold sweats, and blood pressure reduction. A CT scan indicated active bleeding in the peritoneal cavity. This patient immediately received conservative treatments, including blood transfusion therapy and hemostatic drugs. The vital signs of this patient were later stabilized. Other mild complications include pain and abdominal wall edema Eight patients (8.9%) complained of post procedural pain as the local anesthetic subsided. The pain was spontaneously relieved several hours later. Two patients (2.4%) had swelling of the abdominal wall soft tissues after procedure (Fig. 3). which disappeared in 3 days to 1 week.

## Discussion

The peritoneum contains abundant fat, blood vessels, lymph nodes, and connective tissues and fulfills the absorptive and protective functions. There are many types of primary peritoneal lesions, or lesions secondary to those of other organs. The initial manifestation of some abdominal and pelvic malignancies is peritoneal thickening. The patients mainly complain of abdominal discomfort, distension, or pain, which may easily lead to misdiagnosis and missed diagnosis. Radiologic examination alone has limited diagnostic values for peritoneal lesions, while exfoliative cytology of ascites, tumor markers, and other biochemical tests have lower specificity. Therefore, developing a proper method to establish a definite pathologic diagnosis is of high importance.

Laparoscopic inspection is the conventional biopsy method, by which clustered or scattered nodules can be demonstrated on the peritoneal surface under direct vision. This method has a higher accuracy rate, but must be performed in the operating room under general anesthesia. In addition to the high cost, laparoscopic inspection and biopsy may incur such complications as air embolism and intestinal injury [4]. Percutaneous needle biopsy, which is easier to implement, serves as an alternative to laparoscopy [5]. In the present study, CT-guided percutaneous fine-needle aspiration biopsy was performed for peritoneal lesions. The specimens were successfully collected for histopathologic examination, and the accuracy rate was 95.1%, which was consistent with laparoscopy (93.1%), as previously reported [6].

Ultrasound-guided needle biopsy is more suitable for superficial peritoneal lesions [7]. However, this method is more easily disturbed by intestinal gas in the presence of nodular peritoneal thickening or deep-lying peritoneum. This makes it difficult to differentiate between the peritoneum and intestinal canal and restricts its application [8]. The implementation of ultrasound-guided biopsy requires extra caution and the use of a high-frequency probe to acquire the echoic patterns of the thickened peritoneum and to detect the possible presence of nodules [9]. Furthermore, ultrasound-guided biopsy has a narrower scope of indications. Among patients with a large quantity of ascites, the ascites should be treated first; the biopsy cannot be performed until the ascites reduces. Otherwise, ultrasound-guided biopsy is very likely to cause postoperative bleeding, for which hemostasis may be difficult [7]. CT-guided percutaneous needle biopsy was first used in 1976, and is considered a safe and accurate diagnostic method [1]. CT-guided percutaneous needle biopsy has high spatial and density resolution, and can clearly visualize the lesions and cross-sectional anatomy of surrounding tissues. This method has already been applied to the chest, abdomen, and musculoskeletal system [10] (especially lung biopsies), but few reports have been available for use in peritoneal lesions. In this study, a cutting biopsy needle was used, which allowed for multi-angle repeated biopsies. This procedure is easy to perform, has a high accuracy rate, and fewer complications. It is of special value for the diagnosis of peritoneal lesions, especially the differential diagnosis of carcinomatous and tuberculous peritonitis.

Tuberculous and carcinomatous peritonitis are the most common types of peritoneal lesions encountered in the clinic. Tuberculous and carcinomatous peritonitis accounted for 17.3% (14/81) and 75.3% (61/81) of the lesions in the present study, respectively. The clinical manifestations of the two types of lesions overlap, and the differentiation is difficult when the primary lesions are unidentified. Tuberculous peritonitis is a diffuse infectious disease, which mainly occurs in young women, and the incidence is rising every year [11]. Upon CT examination, tuberculous peritonitis generally presents as a small amount of ascites, smooth and thickened parietal peritoneum, as well as lymph node enlargement and calcifications. These findings represent the high diversity of pathologic types [12]. Exudates are produced by an early immune response. After the exudates are absorbed, fibrous hyperplasia, linear-shaped and asteriated changes of the mesentery and multiple large nodules may occur. Further thickening may finally result in an omental cake. The conventional method for confirming tuberculous peritonitis requires identification of acid-fast bacilli in the ascites; however, the positive rate of smears is usually low and ascites culture is time-consuming, which makes it not suitable for early diagnosis [13]. Among patients with tuberculous peritonitis in the present study, one patient was misdiagnosed with late-stage ovarian cancer before surgery. Both pathologic examinations indicated tuberculous peritonitis. A similar finding has been reported in the literature, in which the clinical symptoms of tuberculous peritonitis resembled those of late-stage ovarian cancer [14].

Peritoneal lesions vary greatly in morphology and position and are usually combined with different degrees of ascites. The puncture route should be designed based on the anatomic position of the lesions. If the lesions are located in the adjacent abdominal wall, then the biopsy can be directly performed via the route away from the intestinal canal. If the lesions lie deep in the peritoneal cavity, the route for needle insertion will be relatively narrow, and A coaxial needle can be used. For the latter, the blunt needle core is inserted step-by-step under CT guidance, pushing the intestinal canal to expose the lesion. Any deviation of puncture direction should be timed corrected to maximally reduce organ damage [15]. In the present study, 11.9% (10/84) of the patients had coaxial needle biopsies. If the relationship between the lesion and adjacent blood vessels and intestinal canal is uncertain, then the puncture route should be designed based on historical images of contrast-enhanced CT scan. Cutting will be performed on the tissue surface. If the specimens are too short, sampling can be repeated several times in an attempt to avoid damage to the intestinal canal.

The accuracy rate of CT-guided percutaneous fine-needle aspiration biopsy in the present study was 95.1%. Errors might arise from the following: (1) Patient breathing movements differ, and even a tiny angular error will alter the puncture route. CT cannot dynamically monitor the lesion position, and localization cannot ensure the accuracy of the puncture site. (2) Some lesions were relatively thin and showed a nodular scattered distribution. As the route was narrow and the biopsy specimens were smaller and fragmented, the specimens might be insufficient for pathologic examination. (3) The peritoneum at the puncture site only had exudative changes in some patients, and the biopsy specimens did not contain the primary or secondary peritoneal lesions. With respect to complications, only 1 patient had intra-abdominal bleeding, which was controlled with conservative treatment. The reasons may include the following: patients did not breathe as required, and the operation was not fast enough. As a result, the blood vessels were damaged by needle cutting [1]. Therefore, while observing the breathing of patients, the operation should be implemented fast and deftly, with fast needle insertion and sampling along the margin of lesions, and the operative time should be reduced as much as possible. Another two patients had ascites leakage and swelling of subcutaneous soft tissues after surgery, which are common complications of such a procedure [16]. The reasons may be due to the thin fat layer in the abdominal wall, poor immunity, and low sealing performance of conventional sterile gauze. As a countermeasure, the abdominal wall can be wrapped with elastic bandage for a week or subjected to pressurized immobilization for 24 h to reduce the risk of infection.

The present study had certain limitations. First, as the treatment scheme is only developed based on pathologic results, patients with peritoneal metastases accounted for a larger proportion in this study, while those with benign lesions only accounted for a small proportion. Second, three patients were lost to follow-up, and no definite diagnosis was made due to the lack of pathologic or clinical follow-up data. Third, as CT scan uses radiation, CT guidance was only used when ultrasound guidance was insufficient for peritoneal thickening, which inevitably led to a small sample size.

## Conclusion

To conclude, CT-guided percutaneous fine-needle aspiration biopsy has high sensitivity and safety for peritoneal lesions. This procedure not only informs etiologic identification, but also contributes to subsequent treatment and prognostic prediction.

## Data Availability

The datasets used or analysed during the current study are available from the corresponding author on reasonable request.
